# Dialing up desire and dampening disinterest: regulating sexual desire in the bedroom and sexual and relationship well-being

**DOI:** 10.1177/02654075211054781

**Published:** 2021-11-26

**Authors:** Rebecca M. Horne, Stephanie Raposo, Amy Muise, Cheryl Harasymchuk, Emily A. Impett

**Affiliations:** 1 7938 University of Toronto, Toronto, ON, Canada; 2 7991 York University, Toronto, ON, Canada; 3 6339 Carleton University, Ottawa, ON, Canada; 4 71637 University of Toronto Mississauga, Toronto, ON, Canada

**Keywords:** sexual desire regulation, suppression, amplification, authenticity, sexuality, romantic relationships

## Abstract

Romantic partners often regulate their emotions and affection to achieve certain goals, but research has yet to explore how partners regulate their expression of sexual desire during sex and its implications for couples’ well-being. In two multi-part dyadic diary studies of primarily mixed-gender couples in longer-term relationships residing in North America, we examined three questions. First, is amplifying desire and suppressing disinterest during sex associated with both partners’ daily sexual and relationship satisfaction? Second, do these associations differ by level of sexual desire and gender? Third, tested in our second sample, can these associations be explained by feelings of sexual inauthenticity? Across both samples (N_total_ = 225 couples, 450 participants), amplifying desire was associated with lower sexual satisfaction, while suppressing disinterest was not associated with daily satisfaction. Importantly, sexual desire played a role in the links between desire regulation during sex and satisfaction: on days when people were low in sexual desire, amplification was associated with both partners’ lower sexual satisfaction, while suppression was associated with a partner’s higher relationship satisfaction. In addition, amplification (on low desire days) and suppression (regardless of desire level) were associated with lower sexual authenticity which, in turn, was linked to lower relationship satisfaction. The findings suggest that desire regulation during sex plays an important role in couples’ daily sexual satisfaction and relationship satisfaction—in part because it feels sexually inauthentic—with the implications of this regulation being particularly strong when people feel low sexual desire.

Expressions of sexual interest and desire are often expected in romantic relationships (Bennett & Denes, 2019). Because pleasing a romantic partner is a strong motivator to engage in sex (Impett et al., 2005; Meston & Buss, 2009), people often want to see that their partner desires and is enjoying a sexual experience. However, not all sexual interactions are strongly desired (Impett & Peplau, 2003; O’Sullivan & Allgeier, 1998). Given that people may experience levels of sexual desire that are incongruent with the interest they wish to express to their partner during sex, they may attempt to regulate their expressions of sexual desire. For instance, people might feel some desire for their partner during sex but wish to express more desire than they currently feel (e.g., through erotic talk, caressing) to show love for their partner. On the other hand, people might lose interest during a sexual encounter because their partner is not attending to their needs but conceal this frustration by not disclosing these feelings. This begs the question: is such desire regulation in the bedroom associated with better or worse sexual well-being and relationship quality? In the current research, we extend emotion regulation theory and research on deceptive affection in romantic relationships to the domain of sexuality to test whether *amplifying sexual desire* (i.e., exaggerating the expression of desire) and *suppressing sexual disinterest* (i.e., concealing feelings of low desire) during sex are associated with sexual and relationship satisfaction in daily life.

## Emotion Regulation and Deceptive Affection in Romantic Relationships

Sexual desire is generally defined as a motivation to seek out and engage in sexual experiences, but it has also been classified as an emotional state and overlaps with definitions of affection (Gonzaga et al., 2006, 2008). As such, our examination of sexual desire regulation during sex is grounded in literature from affective science (emotion regulation) and communication (deceptive affection). *Emotion regulation* involves influencing the timing, experience, and expression of emotions to achieve personally relevant goals (Gross, 2015). Of particular interest in interpersonal contexts are response-focused strategies that aim to alter the expression of an emotion after it is elicited. Response-focused strategies present a tension between expression and experience of an emotion: an individual can control which emotion their partner sees, but they also tend to be relatively less successful in shifting their inner experience toward that desired emotion (English et al., 2013). Amplification (exaggerating emotional display; Côté & Morgan, 2002) and suppression (inhibiting emotional display; Gross & John, 2003) are two response-focused strategies with negative relational consequences documented in several interpersonal contexts (e.g., with romantic partners, acquaintances, colleagues, children; Côté & Morgan, 2002; English & John, 2013; Impett et al., 2012; 2014; Le & Impett, 2016).

In addition to regulating emotions, people also regulate the expression of other internal processes. For example, research finds that *deceptive affection*—expressing affection (e.g., fondness, warmth) to a partner that is incongruent with one’s actual feelings—is a common occurrence in romantic relationships (reported on average three times per week; Horan & Booth-Butterfield, 2013). People may amplify or suppress their affection for varying reasons (e.g., to maintain a relationship or hide negative feelings; Denes et al., 2017). Although people who recalled engaging in deceptive affection to a partner showed similar levels of physiological arousal and negative emotion (e.g., guilt) compared to those who recalled engaging in honest affection (Horan & Booth-Butterfield, 2011), little work has assessed the *relational* outcomes of regulating affection expression (e.g., Bennett & Denes, 2019; Denes et al., 2017; Gillen & Horan, 2013) or how these links might extend to the regulation of sexual desire.

Research on emotion regulation and deceptive affection are relevant to desire regulation for several reasons. Although these literatures examine distinct—but related—phenomena (i.e., emotion vs. affection), both focus on incongruence between feeling and expression and the implications of regulating outward expression for romantic relationship quality—a focus applicable to the domain of sexuality and the incongruence between feelings and expressions of desire. In addition, both bodies of literature identify amplification and suppression as important regulation strategies in interpersonal contexts, which further supports our questions about how these strategies shape the way that sexual desire is expressed to a partner during sex.

## Sexual Desire Regulation During Sexual Encounters

Sex is a domain of relationships in which people have difficulty communicating to a partner (Byers, 2011; Rehman et al., 2017), but also a domain with especially strong expectations for positive expressions (e.g., affection, interest; Bennett & Denes, 2019). During sex, people might be particularly motivated to express high desire, sometimes even feigning or exaggerating such feelings during consensual sexual experiences (Impett & Peplau, 2003; O’Sullivan & Allgeier, 1998). In one study, 69% of women and 57% of men reported faking enthusiasm or arousal during sex; further, 50% of women and 25% of men reported feigning an orgasm (Muehlenhard & Shippee, 2010), often to protect a partner’s feelings or to end a sexual experience when a real orgasm was unlikely (Muehlenhard & Shippee, 2010; Séguin et al., 2015). People who pretended to orgasm during sex, compared to those who had authentic orgasms, also reported less trust, closeness, and commitment (Denes et al., 2019). While positive self-disclosure and erotic talk during sex predict higher satisfaction (Denes et al., 2020), deceptive affection (operationalized as amplifying and suppressing affection) during sex was associated with lower sexual and relationship satisfaction (Bennett & Denes, 2019).

In addition to exaggerating feelings of desire, people might also strive to conceal feelings of sexual disinterest. Babin (2013) found that people who experienced anxiety with sexual communication and viewed themselves as unskilled sexual partners engaged in fewer verbal and non-verbal expressions of pleasure during sex. Fewer non-verbal (but not verbal) pleasure displays were, in turn, associated with lower sexual satisfaction (Babin, 2013), suggesting that low desire expression—or even disinterest suppression—may undermine sexual well-being. On the more extreme end, about 30% of women reported pain during their last sexual experience, but less than half (43%) of them told their partner (Herbenick et al., 2015). Our work integrates and extends these findings by investigating whether desire regulation during sex is associated with lower sexual and relationship satisfaction in daily life. While our primary focus is on associations among desire regulation during sex and one’s *own* satisfaction, it is also possible that desire regulation could be linked to a *partner’s* daily outcomes. If people can detect that their partner is regulating desire during a sexual encounter, then they might feel less satisfied knowing that their partner’s behavior is misaligned with their inner feeling. But to the extent that people can convincingly exhibit the level of desire they wish to portray during sex, then partners may not recognize regulation is occurring, thus protecting the partner’s satisfaction. We only know of one study that examined whether emotion suppression during a sexual encounter predicted a partner’s relationship quality in a clinical sample of women with low sexual desire (Dubé et al., 2019), but no evidence of partner effects for suppression was found. Given the conceptual plausibility of such partner effects—but limited empirical evidence—we explore the possibility that one’s desire regulation during sex is also associated with a partner’s sexual and relationship outcomes.

## The Moderating Role of Sexual Desire

To our knowledge, research has yet to examine how relevant contextual factors may shape the association between regulation during sex and partners’ sexual and relationship well-being. When considering desire regulation, a person’s *general level of sexual desire* for their partner on a given day might be an especially important factor to consider. Response-focused regulation strategies such as amplification and suppression present a disconnect between one’s displayed and felt emotion (English et al., 2013; Gross, 2015). Gaps between emotional expression and inner experience may induce feelings of incongruence or self-discrepancy, which is linked to lower psychological well-being (e.g., negative emotions)—especially when the magnitude of that discrepancy is high (Higgins, 1987). In other words, the greater the discrepancy between an individual’s general level of sexual desire on a given day and their regulated desire during sex, the lower their satisfaction may be. As such, amplifying desire during sex on days when people generally lack desire for their partner might feel more distressing for one’s relationship appraisals than amplifying desire during sex on days when people feel moderate or high levels of desire for their partner. Likewise, if people feel low desire for their partner on a given day, suppressing disinterest during sex may have an even stronger negative association with satisfaction. Indeed, a recent study found that when women coping with clinically low levels of sexual desire (or their partner) suppressed negative emotions during sexual interactions, they reported lower personal and relationship well-being (Dubé et al., 2019). We investigate the nuance in how desire regulation during sex plays out in daily life by isolating its associations with sexual and relationship satisfaction on days when people feel relatively less or more sexual desire for their partner.

## The Mediating Role of Sexual Authenticity

One key reason that desire regulation during sex may be linked to poorer sexual and relationship well-being—especially on days when one’s general level of sexual desire for their partner is low—is because it may be tied to feelings of low *authenticity* during the sexual encounter in which regulation is occurring. Authenticity involves behaving in ways that are consistent with one’s thoughts, feelings, and core sense of self (Kernis & Goldman, 2006) and has been linked to a host of positive outcomes (e.g., lower anxiety and depression, greater relationship and life satisfaction; Kernis & Goldman, 2006; Sheldon et al., 1997). Importantly, expressing emotions in ways that are discordant from one’s genuine feelings leads people to feel and be perceived as *inauthentic*, which may have negative relational consequences (Impett et al., 2012, 2014; Sheldon et al., 1997). People may be particularly vulnerable to feeling inauthentic during amplification and suppression because these regulation strategies alter the expression, but not the experience, of emotions (Gross, 2015). For example, suppressing negative emotions while sacrificing for a romantic partner has been linked to feeling and being perceived by a partner as less authentic compared to not suppressing emotions, and in turn lower daily relationship quality (Impett et al., 2012, 2014). Likewise, Bennett and Denes (2019) suggested that one reason deceptive affection during sex is associated with lower satisfaction is because it draws one’s attention to experiencing incongruence between felt and expressed affection. Thus, we test whether one reason that desire regulation during sex is linked to lower daily relational and sexual well-being is because it is associated with feelings of sexual inauthenticity.

## Gender Differences in the Link Between Sexual Desire Regulation and Outcomes

Desire regulation during sex may also differentially predict partners’ relationship and sexual well-being depending on one’s gender. Although men report using more deceptive affection in their romantic relationships than women (Horan & Booth-Butterfield, 2013) and feel less guilt and shame after doing so (Horan & Booth-Butterfield, 2011), women feign orgasm more often than men (Muehlenhard & Shippee, 2010). Nevertheless, other work found no associations between gender and deceptive affection during sex (Bennett & Denes, 2019). Gender may, however, play an important role in desire regulation in light of traditional sexual scripts. Governed by sociocultural gender norms, these scripts suggest that heterosexual men and women are exposed to different rules for how they ought to approach sexual interactions (e.g., men “should” always desire and be ready for sex, women “should” resist sexual advances or prioritize emotional experience; Masters et al., 2014). It is possible that men may feel especially distressed when they regulate desire and experience lower satisfaction compared to women, who may be more practiced in (and perhaps less influenced by) desire regulation during sex.

## Overview of the Current Study

In two 21-day dyadic experience studies of primarily long-term, mixed-gender couples, we investigated three research questions. Given that we have the same measures across studies for the first two research questions, we combined data from two separate datasets and tested these questions using integrative data analyses (IDA; Curran & Hussong, 2009; Hussong et al., 2013). First, does desire regulation (i.e., amplifying sexual desire or suppressing sexual disinterest) during sex predict sexual and relationship satisfaction in daily life? We predicted that both forms of desire regulation would be associated with one’s own lower sexual and relationship satisfaction and explored the possibility that they may also be associated with a partner’s satisfaction. Second, are amplification and suppression during sex differentially associated with satisfaction depending on a person’s level of sexual desire for their partner or by their gender? We predicted that negative links between desire regulation and satisfaction would be stronger on days when individuals had low sexual desire for their partner and that if gender differences emerged, desire regulation would be more strongly linked to lower satisfaction for men compared to women. Finally, does sexual authenticity mediate links between desire regulation during sex and daily satisfaction? In our second sample, we tested the prediction that desire regulation would be linked to lower sexual authenticity which, in turn, would predict lower daily satisfaction.

## Method

### Participants and Procedure

#### Sample 1

We recruited 122 couples to participate in a study on relationship experiences through online advertisements on Reddit and Kijiji (posted in five major Canadian cities) and posters in one major Canadian city. Our sample size was determined by similar experience sampling studies in our labs in which we aimed to recruit at least 120 couples. Couples had to be in an exclusive, monogamous relationship for at least 2 years, living together, and both partners had to agree to participate. Participants were pre-screened for eligibility and after consenting to participate, both partners were emailed a unique link to a baseline survey (55 minutes), and then for the next 21 consecutive days, a brief survey (10–15 minutes). Participants completed the surveys before bed but had access to the surveys between 5:00 p.m. and 9:00 a.m. the next morning. Three months following the last daily survey, participants completed a follow-up survey (20–30 minutes). Each partner was paid up to $55 CAD. Participants completed 4773 daily surveys in total (*M* = 19.56 per person).

In terms of self-identified gender, the sample consisted of 125 women, 111 men, and one transgender woman (six people did not identify their gender).. Participants were 31.53 years old on average (*SD* = 9.46), and most (83.6%) identified as heterosexual, followed by lesbian (6.1%), bisexual (4.5%), bicurious (0.8%), gay (0.8%), queer (0.8%), and pansexual (0.4%). In terms of ethnicity, participants identified as White/European (76.2%), Latin American (6.6%), Bi-/Multi-Ethnic (4.9%), East Asian (4.1%), South Asian (2.5%), and Black (2.0%). Couples were together for 8.42 years on average (*SD* = 7.10), and 56.2% were married, 22.2% were dating, and 21.6% were engaged. Most (75.0%) participants did not have children.^1^

#### Sample 2

We recruited 121 couples through several websites (e.g., Facebook, Reddit), community postings, and the research team’s study database with the same procedure as in Sample 1 guiding our sample size target. Eligible participants had to be at least 18 years old, in a romantic relationship, sexually active, living together or seeing each other at least five out of 7 days per week, residing in Canada or the United States, able to read and understand English, have daily access to a computer with internet, and both partners had to agree to participate. Eligibility and relationship status were confirmed through telephone interviews. After obtaining informed consent, participants completed a 30- to 45-minute baseline survey, brief (5- to 10-minute) daily surveys for 21 consecutive days, and a (20-minute) 3-month follow-up survey. Each participant was compensated up to $60 CAD. Participants completed 4488 daily surveys in total (*M* = 18.39 per person).

The sample consisted of 124 women, 115 men, and two people who identified as “other” (one person did not identify their gender). Participants were 32.63 years old on average (*SD* = 10.19), and most (81.4%) identified as heterosexual, followed by bisexual (9.1%), asexual (2.9%), lesbian (2.5%), pansexual (1.7%), gay (0.8%), queer (0.8%), and “other” (0.8%). In terms of ethnicity, participants identified as White/European (65.3%), East Asian (8.3%), South Asian (7.4%), Bi-/Multi-Ethnic (5.8%), Black (4.5%), Latin American (4.1%), and “other” (4.1%). Couples were together for 8.50 years on average (*SD* = 8.41), and most were married (46.7%), cohabiting (29.3%), or common law (13.6%). Two-thirds (68.6%) of participants did not have children.^2^

### Daily-Level Measures

Across both samples, we assessed daily measures with only a few items or a single item to increase efficiency and minimize participant attrition (Bolger et al., 2003). ***Sexual satisfaction*** was assessed with five items from the Global Measure of Sexual Satisfaction (GMSEX; Lawrence & Byers, 1995) adapted to the daily context and rated on a semantic differential scale from 1 to 7 (e.g., “bad” to “good”; *M* = 5.69, *SD* = 1.62; Sample 1 *Rc* = .90; Sample 2 *Rc* = .96)^3^. ***Relationship satisfaction*** was assessed with one item adapted from the Perceived Relationship Quality Components (PRQC) Inventory (Fletcher et al., 2000) to ask about that day: “How satisfied were you with your relationship?” (1 = “not at all” to 7 = “extremely”; *M* = 6.09, *SD* = 1.21). ***Sexual desire*** was assessed with one item: “I felt a great deal of sexual desire for my partner today” (1 = “strongly disagree” to 7 = “strongly agree”; *M* = 4.59, *SD* = 1.79).

Each day, participants were also asked “Did you and your partner have sex today? (yes/no).” Across both samples, participants reported engaging in sex with their partner on a total of 1937 days (21.03% of days; *M* = 3.99, *SD* = 3.09). A final sub-sample of 225 couples were included in our analyses because 18 couples did not have sex during the diary. Each day participants reported engaging in sex, ***amplifying sexual desire*** was assessed with an item adapted from Côté and Morgan (2002): “During sex, I tried to enhance or exaggerate my display of sexual desire” (1 = “strongly disagree” to 7 = “strongly agree”; *M* = 2.58, *SD* = 1.83); ***suppressing sexual disinterest*** was assessed with an item adapted from Gross and John (2003): “When I felt disinterested during sex, I was careful not to express this” (1 = “strongly disagree” to 7 = “strongly agree”; *M* = 2.54, *SD* = 1.91); and in Sample 2 only, ***sexual authenticity*** was assessed with one item adapted from Impett et al. (2012) to the sexual context: “I felt authentic (true to myself) during sex” (1 = “strongly disagree” to 7 = “strongly agree”; *M* = 6.16; *SD* = 1.15).

### Data Analytic Approach

We analyzed the data with multilevel modeling and the Actor Partner Interdependence Model (APIM; Kenny et al., 2006) using mixed models in SPSS 27.0. We tested two-level cross-classified models with random intercepts and random slopes for all within-person main effects in which persons are nested within dyads, and person and days are crossed to account for the fact that both partners completed the daily surveys on the same days (Kenny et al., 2006). If a model did not converge with random slopes, we inspected the estimates of covariance parameters to determine which effects were contributing to the error (i.e., effects with zero random variability), removed these effects from being estimated as random until the model converged, and retained this more parsimonious model. Only one model converged when estimating random slopes—but the effects in this model changed negligibly if estimated with or without random slopes^4^—and all others would not converge, so we retained fixed effects only models. Daily predictors (i.e., desire amplification and disinterest suppression during sex) and the moderator (i.e., sexual desire) were partitioned into their within- and between-variance components, which were person-mean centered and aggregated over the course of the diary, respectively (Raudenbush et al., 2004).

Given that we had the same measures in two datasets to answer our first two research questions and sought to find more robust effects across samples, we conducted an integrative data analysis (IDA) following recommendations by Curran and Hussong (2009) and Hussong et al. (2013). We pooled these datasets together, computed an effect-coded sample variable (Sample 1 = 1; Sample 2 = −1), and interacted this variable with all terms in our models. If there were no significant interactions with sample, then following guidelines on model trimming (Curran & Hussong, 2009; Hussong et al., 2013), we removed these interactions to create a more parsimonious model while retaining the main effect for sample. If there were significant interactions with sample, then we ran simple effects tests with a dummy-coded sample variable (first Sample 1 = 1 and Sample 2 = 0, then Sample 1 = 0 and Sample 2 = 1) to probe the interaction effect within each sample. We first ran two models with the main effects of both desire regulation variables^5^—including actor, partner, within-person, and between-person effects—predicting sexual satisfaction and relationship satisfaction. In each of these models, we then tested moderation by sexual desire. If there was a significant interaction term between each of the desire regulation variables and sexual desire, simple effects were estimated at one standard deviation (*SD*) above and below the mean of sexual desire to represent high and low levels of sexual desire, respectively. We ran all models as indistinguishable, but tested whether gender moderated the effects (e.g., three-way interactions among amplification during sex, sexual desire, and gender). If an effect differed by gender, we ran distinguishable models and reported the effects separately for men and women. Although our focus was on whether within-person daily changes in desire amplification and disinterest suppression were associated with daily sexual and relationship satisfaction, as well as if these effects were moderated by daily sexual desire, between-person effects are included in the tables for interested readers.

Finally, in Sample 2 only, we tested mediated-moderations (Muller et al., 2005) to see if sexual authenticity accounted for the links between the desire regulation and sexual desire interactions on relationship and sexual satisfaction. We used the Monte Carlo Method for Assessing Mediation with 20,000 resamples and 95% confidence intervals (MacKinnon et al., 2004) and concluded that the indirect effect of the desire regulation and sexual desire interaction on satisfaction through authenticity was significant if zero was not in the 95% confidence interval. Following Muller et al.’s (2005) procedure and our conceptual model, we tested whether sexual desire moderated links between desire regulation and sexual authenticity. If the interaction between desire regulation and sexual desire did not predict sexual authenticity, we interpreted the direct effect of desire regulation on sexual authenticity. In line with our daily models, we ran full APIMs^6^, with separate models for the two dependent variables. Truncated data and code are available on the OSF (https://osf.io/w7gnv/?view_only=1e18eb4c94424e8da8644165a70c5de8). Correlations among all variables are shown in Table 1.^7^

**Table 1.** Correlations Among Study Variables Across Samples (n = 486).

Variable	1	2	3	4	5	6
1. Daily Sexual Desire	.40***					
2. Daily Desire Amplification During Sex	−.09^†^	.36***				
3. Daily Disinterest Suppression During Sex	−.14**	.61***	.21***			
4. Daily Relationship Satisfaction	.52***	−.26***	−.26***	.56***		
5. Daily Sexual Satisfaction	.39***	−.27***	−.22***	.57***	.57***	
6. Gender	.22***	−.05	−.01	−.04	.02	–

*Note.* Gender coded as 0 = women, 1 = men. Partner correlations bolded on the diagonal. Daily variables aggregated across the course of the diary. *** *p* < .001, **p < .01, *p < .05, ^†^*p* = .053.

## Results

Throughout this section, we first report findings from the models that only contain the main effects of desire regulation during sex on satisfaction, and then report whether sexual desire moderated these effects.

### Main Effects of Desire Regulation During Sex

Consistent across samples, sexual desire amplification during sex predicted lower sexual satisfaction (*b* = −.04, *SE* = .01, *t*(1194.80) = −3.02, *p* = .003, 95% CI [−.07, −.02]). There was a significant interaction between actor sexual disinterest suppression during sex and sample (*b* = −.03, *SE* = .01, *t*(1198.46) = −2.63, *p* = .009, 95% CI [−.06, −.01]—as well as between partner sexual disinterest suppression during sex and sample (*b* = −.03, *SE* = .01, *t*(1198.47) = −2.08, *p* = .038, 95% CI [−.05, −.002])—predicting sexual satisfaction. In Sample 1, neither actor (*b* = .00, *SE* = .02, *t*(1192.08) = .20, *p* = .843, 95% CI [−.03, .04]) nor partner (*b* = .02, *SE* = .02, *t*(1192.08) = .99, *p* = .322, 95% CI [−.02, .05]) suppression were linked to sexual satisfaction. In Sample 2, however, actor suppression was linked to lower sexual satisfaction (*b* = −.07, *SE* = .02, *t*(1202.80) = −3.08, *p* = .002, 95% CI [−.11, −.02]), and partner suppression was linked to marginally lower actor sexual satisfaction (*b* = −.04, *SE* = .02, *t*(1202.80) = −1.79, *p* = .073, 95% CI [−.08, .004]). There were no significant associations among either form of desire regulation during sex and daily relationship satisfaction, as well as no gender differences in the links between desire regulation and sexual or relationship satisfaction.

### Interactions With Sexual Desire

In both samples, there was a significant interaction between actor amplification and actor sexual desire—as well as between partner amplification and partner sexual desire—predicting sexual satisfaction (Table 2). On days when sexual desire was low, desire amplification during sex was linked to lower actor (*b* = −.12, *SE* = .04, *t*(1210.64) = −3.37, *p* = .001, 95% CI [−.19, −.05]) and partner (*b* = −.11, *SE* = .04, *t*(1210.20) = −3.09, *p* = .002, 95% CI [−.18, −.04]) sexual satisfaction. On days when sexual desire was high, the negative link between amplification and actor sexual satisfaction was attenuated (*b* = −.04, *SE* = .01, *t*(1190.26)=−2.82, *p* = .005, 95% CI [−.07, −.01]), while the link between amplification and partner sexual satisfaction was no longer significant (*b* = −.02, *SE* = .01, *t*(1190.15) = −1.19, *p* = .233, 95% CI [−.05, .01]). There was also a significant three-way interaction among actor suppression, actor desire, and sample. However, when we probed this three-way interaction, the two-way interaction between actor suppression and sexual desire was non-significant in Sample 1 (*b* = .01, *SE* = .01, *t*(1222.63) = .71, *p* = .477, 95% CI [−.02, .03]) and marginally significant in Sample 2 (*b* = −.03, *SE* = .02, *t*(1336.46) = −1.77, *p* = .076, 95% CI [−.07, .003]). Gender did not moderate any of these effects.

**Table 2.** Integrative Data Analysis Results: Desire Regulation During Sex and Desire Interactions Predicting Daily Relationship and Sexual Satisfaction Across Samples.

		Actor Daily Relationship Satisfaction				Actor Daily Sexual Satisfaction				
	*b*	*SE*	*t*(df)	*p*	CI	*b*	*SE*	*t*(df)	*p*	CI
**Within-Person Effects**										
A Amplify Desire	−.01	.02	−.28 (1255.67)	.78	[−.05, .04]	−.08	.02	−3.70 (1204.44)	<.001	[−.12, −.04]
A Suppress Sexual Disinterest	.01	.02	.71 (1261.60)	.48	[−.02, .05]	.00	.02	.09 (1218.50)	.93	[−.03, .04]
A Sexual Desire	.19	.02	10.52 (1573.95)	<.001	[.15, .22]	.23	.02	12.30 (1393.71)	<.001	[.19, .26]
P Amplify Desire	−.01	.02	−.67 (1250.79)	.50	[−.05, .03]	−.06	.02	−3.09 (1204.12)	.002	[−.11, −.02]
P Suppress Sexual Disinterest	.03	.02	1.69 (1261.85)	.092	[−.005, .06]	.01	.02	.54 (1214.08)	.59	[−.03, .04]
P Sexual Desire	.07	.02	3.70 (1572.95)	<.001	[.03, .10]	−.02	.02	−1.32 (1390.64)	.19	[−.06, .01]
A Amplify Desire X Desire	.00	.01	.35 (1324.61)	.72	[−.02, .03]	.03	.01	2.25 (1217.96)	.025	[.004, .06]
A Suppress Disinterest X Desire	−.01	.01	−1.24 (1324.84)	.22	[−.04, .01]	−.01	.01	−.90 (1290.13)	.37	[−.04, .01]
P Amplify Desire X Desire	.00	.01	.33 (1291.64)	.74	[−.02, .03]	.03	.01	2.61 (1217.96)	.009	[.01, .06]
P Suppress Disinterest X Desire	−.03	.01	−2.82 (1327.48)	.005	[−.06, −.01]	−.01	.01	−.69 (1243.15)	.49	[−.03, .02]
**Sample Effects**										
Sample	.05	.03	1.60 (161.76)	.11	[−.01, .11]	.08	.05	1.51 (187.91)	.13	[−.02, .18]
A Amplify Desire X Desire X Sample	−.03	.01	−2.88 (1336.85)	.004	[−.04, −.01]					
A Suppress Disinterest X Desire X Sample						−.02	.01	−2.28 (1290.84)	.023	[−.04, −.003]
**Between-Person Effects**										
A Amplify Desire	−.01	.02	−.43 (386.11)	.67	[−.06, .04]	−.02	.04	−.61 (360.34)	.54	[−.09, .05]
A Suppress Sexual Disinterest	−.05	.02	−2.26 (384.51)	.025	[−.10, −.01]	−.12	.03	−3.34 (350.91)	.001	[−.18, −.05]
A Sexual Desire	.27	.03	10.23 (418.95)	<.001	[.22, .32]	.38	.04	9.93 (383.67)	<.001	[.31, .46]
P Amplify Desire	−.02	.02	−.85 (386.16)	.39	[−.07, .03]	−.07	.04	−1.93 (359.96)	.054	[−.14, .001]
P Suppress Sexual Disinterest	−.05	.02	−2.22 (384.44)	.027	[−.10, −.01]	−.01	.03	−.19 (351.23)	.85	[−.07, .06]
P Sexual Desire	.10	.03	3.66 (418.86)	<.001	[.04, .15]	.10	.04	2.59 (380.41)	.010	[.02, .18]
A Amplify Desire X Desire	.03	.02	1.50 (386.14)	.14	[−.01, .06]	.02	.03	.69 (361.99)	.49	[−.03, .07]
A Suppress Disinterest X Desire	−.02	.02	−1.29 (394.38)	.20	[−.06, .01]	−.01	.03	−.27 (362.17)	.79	[−.06, .05]
P Amplify Desire X Desire	−.00	.02	−.26 (386.58)	.80	[−.04, .03]	.03	.03	1.19 (361.08)	.24	[−.02, .09]
P Suppress Disinterest X Desire	−.01	.02	−.81 (394.49)	.42	[−.05, .02]	.01	.03	.45 (362.00)	.65	[−.04, .07]

*Note.* A = actor. P = partner. Amplify Sexual Desire = amplify sexual desire during sex. Suppress Sexual Disinterest = suppress sexual disinterest during sex. Desire = sexual desire. Sample effect coded as 1 = Sample 1, −1 = Sample 2.

Turning to relationship satisfaction, consistent across samples, there was a significant interaction between partner disinterest suppression during sex and sexual desire (Table 2). On days when sexual desire was low, suppression was linked to higher partner relationship satisfaction (*b* = .07, *SE* = .03, *t*(1304.16) = 2.41, *p* = .016, 95% CI [.01, .13]), but on days when sexual desire was high, the link between suppression and partner relationship satisfaction was no longer significant (*b* = −.01, *SE* = .01, *t*(1225.89) = −1.05, *p* = .293, 95% CI [−.04, .01]). There was also a significant three-way interaction among actor amplification, actor desire, and sample. Although the interaction between actor amplification and sexual desire was significant in Sample 1 (*b* = .03, *SE* = .01, *t*(1290.30) = 2.24, *p* = .026, 95% CI [.004, .06]), the simple effects were non-significant. The interaction between amplification and sexual desire was non-significant in Sample 2 (*b* = −.02, *SE* = .02, *t*(1378.52) = −1.15, *p* = .251, 95% CI [−.06, .02]). Gender did not moderate any of these effects.^8^

### Sample 2 Mediations By Sexual Authenticity

The interaction between amplifying desire during sex and sexual desire predicted sexual authenticity, such that on days when people were lower in sexual desire than usual, amplifying desire during sex was associated with lower sexual authenticity (*b* = −.17, *SE* = .06, *t*(750.75) = −2.88, *p* = .004, 95% CI [−.29, −.05]), but on days when people were higher in sexual desire, amplifying desire was not associated with sexual authenticity (*b* = −.02, *SE* = .03, *t*(717.41) = −.88, *p* = .381, 95% CI [−.07, .03]). Sexual authenticity, in turn, was associated with higher daily relationship satisfaction (indirect effect: 95% CI = [.001, .01]), but was not associated with sexual satisfaction (indirect effect 95% CI = [−.001, .01]) (Table 3). Although the interaction between sexual disinterest suppression during sex and sexual desire did not predict daily sexual authenticity, disinterest suppression directly predicted authenticity. On days when people suppressed their disinterest during sex more than usual, they reported lower daily sexual authenticity, which in turn predicted lower relationship satisfaction (indirect effect: 95% CI = [−.01, −.0001]), but not sexual satisfaction (indirect effect: 95% CI = [−.01, .001]).^9^

**Table 3.** Sample 2 Mediation Results: Daily Links Among Desire Regulation During Sex, Desire, and Satisfaction Mediated by Daily Sexual Authenticity.

	**Daily Relationship Satisfaction**					
***Effect***	*b*	*SE*	*df*	*t*	*p*	CI
Amplify Desire X Desire → Sexual Authenticity → DV						**[.001, .010]**
Amplify Desire X Desire (a path)	.05	.02	758.54	2.53	.012	[.012, .094]
Sexual Authenticity (b path)	.09	.03	727.02	3.18	.002	[.033, .140]
Total Effect (c path)	.04	.02	728.31	2.29	.022	[.005, .067]
Direct Effect (c’ path)	.03	.02	723.19	2.04	.042	[.001, .063]
Suppress Sexual Disinterest → Sexual Authenticity → DV						**[−.013, −.0001]**
Suppress Sexual Disinterest (a path)	−.06	.03	748.97	−2.00	.046	[−.122, −.001]
Sexual Authenticity (b path)	.09	.03	727.02	3.18	.002	[.033, .140]
Total Effect (c path)	.00	.02	737.65	.20	.843	[−.040, .050]
Direct Effect (c’ path)	.01	.02	734.18	.46	.644	[−.034, .056]
	**Daily Sexual Satisfaction**					
***Effect***	*b*	*SE*	*df*	*t*	*p*	CI
Amplify Desire X Desire → Sexual Authenticity → DV						[−.001, .007]
Amplify Desire X Desire (a path)	.05	.02	758.54	2.53	.012	[.012, .094]
Sexual Authenticity (b path)	.04	.03	694.19	1.48	.138	[−.014, .102]
Total Effect (c path)	.04	.02	680.72	2.12	.035	[.003, .069]
Direct Effect (c’ path)	.03	.02	686.80	1.89	.059	[−.001, .065]
Suppress Sexual Disinterest → Sexual Authenticity → DV						[−.009, .001]
Suppress Sexual Disinterest (a path)	−.06	.03	748.97	−2.00	.046	[−.122, −.001]
Sexual Authenticity (b path)	.04	.03	694.19	1.48	.138	[−.014, .102]
Total Effect (c path)	.02	.02	692.51	1.02	.309	[−.023, .073]
Direct Effect (c’ path)	.03	.02	698.83	1.06	.290	[−.022, .074]

*Note.* Effects are unstandardized, within-person coefficients. CI = confidence interval. Top row CI in each model represents the indirect effect CI (bolded = significant). DV = dependent variable. Models include both desire regulation during sex variables and control for within-person partner effects, between-person actor effects, and between-person partner effects. Desire = sexual desire.

## Discussion

In two daily experience studies of mostly mixed-gender couples in longer-term relationships residing in North America, we found that regulating the expression of sexual desire through amplifying desire and suppressing disinterest during sex was associated with sexual and relationship satisfaction in daily life. Sexual desire played a role in shaping links among desire regulation during sex and both partners’ satisfaction, with the effects being strongest at low levels of desire. Finally, in Sample 2, sexual inauthenticity was one reason for why associations were found among desire regulation during sex and relationship (but not sexual) satisfaction. By providing a novel integration and extension of research on emotion regulation and deceptive affection in romantic relationships to the domain of sexuality, this study documents the important role of desire regulation during sex for partners’ relationships and sex lives. We found that amplifying desire during sex was associated with lower sexual satisfaction, and when people had low sexual desire for their partner, amplifying desire was linked to *both* partners’ lower sexual satisfaction. Building on existing research that suggests the gap between displayed and felt emotion may feel especially uncomfortable when this incongruence is large (e.g., Gross, 2015; Higgins, 1987), our work suggests that both partners are less satisfied with their sex lives on days when people exaggerate expressions of desire during sex but feel low desire for their partner. In contrast, on high desire days, the negative link between amplifying desire during sex and sexual satisfaction was weaker, and amplifying desire no longer predicted a partner’s sexual satisfaction. In situations when amplifying desire is simply meant to exaggerate desire, rather than conceal low desire, it might not detract from satisfaction. People also tend to feign sexual pleasure (Muehlenhard & Shippee, 2010; Séguin et al., 2015) and use deceptive affection (e.g., Denes et al., 2017; Horan & Booth-Butterfield, 2013) to protect a partner’s feelings or promote their pleasure, so potential prosocial intentions tied to amplifying desire when desire is already relatively high may override harm to people’s relationships or sex lives.

Interestingly, the opposite pattern emerged for suppression and a partner’s relationship satisfaction: when people had low sexual desire for their partner, suppressing disinterest during sex was linked to a partner’s *higher* relationship satisfaction. People may be somewhat effective at suppressing disinterest in the bedroom insofar as they might convince a partner that they are sexually interested and engaged—thus protecting their partner’s satisfaction—even though this concealment may not alter or even have the opposite impact on their own inner desire and satisfaction (see Dubé et al., 2019). Although related research found only moderate accuracy in detecting a partner’s suppression of negative emotions during daily sacrifice (Impett et al., 2014), individuals may be even *less* attuned to their partners’ true feelings during sex compared to other relationship contexts, potentially because suppressors are convincing in the bedroom or because there are more immediate personal goals (e.g., pleasure, orgasm) present. More research is needed on the perceptual cues and accurate detection of suppression during sex.

We also explored whether the links between desire regulation during sex and satisfaction differed for men and women. We anticipated that if gender differences emerged, we would likely see stronger negative associations among desire regulation and satisfaction for men than women given that traditional sexual scripts pressure men to feel and express strong sexual desire (Masters et al., 2014). We did not, however, find consistent evidence for gender differences across our samples. These findings suggest that desire regulation during sex is linked to sexual and relational outcomes in relatively consistent ways for men and women. While some research found gender differences in the frequency of deceptive affection in romantic relationships (Horan & Booth-Butterfield, 2013) and feigning orgasm (Muehlenhard & Shippee, 2010), these differences do not seem to hold for desire regulation in the bedroom and its ties to daily satisfaction—findings that warrant further replication.

In our second sample of couples, we found that amplifying desire (on low desire days) and suppressing disinterest (regardless of desire level) during sex were linked to lower sexual authenticity which, in turn, was linked to lower relationship—but not sexual—satisfaction. Given that we only tested this question in one of our two samples, we offer some tentative conclusions that require further replication. Previous research found that when people engaged in sex for reasons that were discordant with their true values (compared to authentic reasons), they reported lower relationship satisfaction (Brunell & Webster, 2013), and it has been suggested (but not empirically tested) that hyperattention to incongruent feelings is one reason deceptive affection during sex is linked to poorer relational and sexual outcomes (Bennett & Denes, 2019). We extend these findings by showing that amplifying desire during sex may feel *sexually* inauthentic, and in turn, contribute to feeling less satisfied with one’s relationship. Our findings also align with the well-documented negative effects of suppression in romantic relationships (e.g., Dubé et al., 2019; Impett et al., 2012; 2014). Consistent with research that demonstrated inauthenticity mediated the link between suppression and poorer social functioning in close relationships (English & John, 2013), our work suggests that one reason suppressing disinterest during sex specifically is linked to poorer relationship outcomes is because people feel inauthentic during the sexual encounter.

While our integrative data analysis allowed us to uncover several robust findings across our two samples, several links between suppression and satisfaction were only present in one of our samples. In our second sample of couples, we found that suppression was linked to one’s own lower sexual satisfaction, as well as a partner’s lower relationship satisfaction and (marginally) lower sexual satisfaction. These inconsistencies between samples suggest that there are likely times when, or people for whom, desire regulation during sex (and suppression in particular) is harmful, but other times where it might be inconsequential—or even beneficial—for couples’ relationship and sexual well-being. For example, related research on suppression demonstrated that negative emotion suppression during a sacrifice was linked to *higher* personal well-being and relationship quality for those who were highly interdependent (e.g., value harmony in close relationships; Le & Impett, 2013). Similarly, perhaps partners who are highly motivated to meet each other’s sexual needs (i.e., high in sexual communal strength) would be buffered against the negative effects of suppression during sex. Although we extended previous research by examining the role of sexual desire in the link between desire regulation during sex and satisfaction, another important contextual factor may be the type or duration of sex occurring on that day. For example, people who regulate their expressions of desire for a wide array of sexual acts or throughout the entirety of a sexual encounter (e.g., from the time foreplay starts to the time after sex) may feel lower relationship or sexual satisfaction compared to people who regulate their desire only for a particular type of sexual act (e.g., one they do not enjoy as much) or momentarily during the sexual encounter (e.g., in response to a “wrong move” from a partner). Exploring the individual differences and contextual circumstances associated with desire regulation in the bedroom and its associations with partners’ relationships and sex lives would be a fruitful direction for future research.

### Limitations and Future Directions

Several limitations warrant discussion. We assessed each type of desire regulation during sex with one item adapted from more general emotion regulation measures (Côté & Morgan, 2002; Gross & John, 2003), as is common in daily diary studies, to minimize participant attrition (Bolger et al., 2003). Future work would benefit from more nuanced measures of these constructs that specify, for example, the motivation behind amplifying desire and suppressing disinterest. Second, although we tested our questions using ecologically valid study designs, we cannot confirm causality. Future research could experimentally manipulate desire regulation in hypothetical or recalled sexual scenarios (similar to some experimental work in emotion regulation and deceptive affection research; Butler et al., 2003; Horan & Booth-Butterfield, 2011) or assess desire regulation and relationship and sexual outcomes at multiple times throughout the day (akin to multi-wave assessments that can better tease apart temporal dynamics in relationship and sexuality constructs; see Cao et al., 2019; McNulty et al., 2016). Third, our samples were recruited through similar means (e.g., social media posts) and had similar sociodemographic backgrounds (e.g., most couples were mixed-gender, White, married, in longer-term relationships), which limits the generalizability of our findings. For example, couples in newer relationships may experience more novel, exciting sexual interactions that dampen the inclination to regulate desire in the bedroom. As such, when suppressing disinterest or amplifying desire during sex do occur in newer partnerships, these strategies may detract from satisfaction more strongly by signaling potential issues with open expressions of desire in the earliest stages of a relationship. While our study represents the first investigation of two desire regulation strategies during sex, further research could test interrelations among these strategies, relationship quality, and sexual well-being in more diverse, representative samples.

## Conclusion

The current research extends past work on emotion regulation and deceptive affection in romantic relationships to a context in which regulating feelings might be particularly costly: during sex. The findings demonstrate that amplifying desire during sex is linked to lower sexual satisfaction, but suppressing disinterest during sex is not linked to daily satisfaction. However, the implications of desire regulation during sex partially depend on a person’s level of sexual desire on a given day: on low desire days, amplification is linked to both partners’ lower sexual satisfaction, while suppression is linked to a partner’s higher relationship satisfaction. Finally, when people amplify on low desire days or suppress regardless of desire level, they report feeling less authentic during that sexual encounter which, in turn, is linked to lower relationship satisfaction. This research suggests that regulating desire during some of the most intimate moments of romantic partners’ lives plays an important role in their day-to-day sexual well-being and relationship quality, with the bedroom being a salient relationship domain in which people mind the gap between felt and expressed desire.

## Supplemental Material

sj-pdf-1-spr-10.1177_02654075211054781 – Supplemental Material for Dialing Up Desire and Dampening Disinterest: Regulating Sexual Desire in the Bedroom and Sexual and Relationship Well-Being Click here for additional data file. Supplemental Material, sj-pdf-1-spr-10.1177_02654075211054781 for Dialing Up Desire and Dampening Disinterest: Regulating Sexual Desire in the Bedroom and Sexual and Relationship Well-Being by Rebecca M. Horne, Stephanie Raposo, Amy Muise, Cheryl Harasymchuk and Emily A. Impett in Journal of Social and Personal Relationships

## Notes

1 See Table S1 in supplement for full sample demographics.

2 See Table S2 in supplement for full sample demographics.

3 While the measure of sexual satisfaction was included on all diary days in Sample 2, in Sample 1, participants were only presented with measures of sexual satisfaction on days when they indicated having sex with their partner. We were unable to compute a within-person reliability (indicated by *Rc*) for sexual satisfaction in the combined sample, which may have been due to the discrepancy between the days that it was assessed across the two samples.

4 See Table S3 in the supplement for this specific model with random slopes.

5 We tested the within-person correlation between desire amplification and disinterest suppression during sex using the rmcorr package in R (Bakdash & Marusich, 2017) and found that *r* = .34 in Sample 1 and *r* = .28 in Sample 2. As such, we included both desire regulation variables in the same model.

6 In line with our conceptual model, to isolate the effects of the regulator's own authenticity, we ran our mediated-moderation models without partner authenticity main effects and interactions.

7 See supplement for correlations and descriptive statistics within each sample.

8 Research suggests that the effects of chronically regulating feelings may manifest more strongly over time (e.g., Denes et al., 2017; Gross & John, 2003). As such, we also examined associations among desire regulation during sex and relationship quality 3 months later. We used the aggregates of participants' daily desire amplification and disinterest suppression over the course of the diary to predict follow-up reports of relationship satisfaction, commitment, and break-up thoughts (plus sexual satisfaction in Sample 2), controlling for the same outcome at baseline. The most robust effect from the integrative data analysis revealed that consistent across samples, people who chronically suppressed disinterest during sex reported lower relationship satisfaction and commitment 3 months later. Full details from these analyses are in the supplement.

9 To establish further empirical support for our proposed mediated-moderation model, we tested alternative mediation models (i.e., with sexual authenticity as the dependent variable and as the independent variable) to rule out other pathways. Importantly, we found the strongest support for our predicted model. See supplement for details.
