# Supplementary material for: Dialing up desire and dampening disinterest: regulating sexual desire in the bedroom and sexual and relationship well-being
Source: J Soc Pers Relat. 2021 Nov 26;39(6):1551–73. doi: 10.1177/02654075211054781 (PMC9092913; doi:10.1177/02654075211054781)
Supplement: sj-pdf-1-spr-10.1177_02654075211054781 – Supplemental Material for Dialing Up Desire and Dampening Disinterest: Regulating Sexual Desire in the Bedroom and Sexual and Relationship Well-Being [file sj-pdf-1-spr-10.1177_02654075211054781.pdf]

Supplemental Materials

Dialing Up Desire and Dampening Disinterest: Regulating Sexual Desire in the Bedroom and Sexual and Relationship Well-Being

Table S1

Sample 1 Demographics (n = 244)

Demographic Variable	*M* (range) or *n*	*SD* or %
Age (years)	31.53 (19 - 67)	9.46
Gender		
Women	125	51.2%
Men	111	45.5%
Transgender Woman	1	.4%
Sexual Orientation		
Heterosexual	204	83.6%
Lesbian	15	6.1%
Bisexual	11	4.5%
Gay	2	.8%
Queer	2	.8%
Asexual	1	.4%
Bicurious	1	.4%
Pansexual	1	.4%
Ethnicity		
White/European	186	76.2%
Latin American	16	6.6%
Bi-/Multi-Ethnic	12	4.9%
East Asian	10	4.1%
South Asian	6	2.5%
Black	5	2.0%
Relationship Status		
Married	137	56.2%
Dating (exclusive)	54	22.2%
Engaged	53	21.6%
Relationship Duration (years)	8.24 (2 - 48)	7.10
Children Living at Home		
0	183	75.0%
1	29	11.9%
2	21	8.6%
3	4	1.6%

Note. Percentages may not add up to 100% due to a small amount of missing data.

Table S2

Sample 2 Demographics (n = 242)

Demographic Variable	*M* (range) or *n*	*SD* or %
Age (years)	32.63 (20 - 78)	10.19
Gender		
Women	124	51.2%
Men	115	47.5%
Other	2	.8%
Sexual Orientation		
Heterosexual	197	81.4%
Bisexual	22	9.1%
Asexual	7	2.9%
Lesbian	6	2.5%
Pansexual	4	1.7%
Gay	2	.8%
Queer	2	.8%
Other	2	.8%
Ethnicity		
White	158	65.3%
East Asian	20	8.3%
South Asian	18	7.4%
Bi-/Multi-Ethnic	14	5.8%
Black	11	4.5%
Latin American	10	4.1%
Other	10	4.1%
Relationship Status		
Married	113	46.7%
Engaged	19	7.9%
Dating	3	1.2%
Cohabiting	71	29.3%
Common-Law	33	13.6%
Other	1	.4%
Relationship Duration (years)	8.50 (1.5 - 58.5)	8.41
Children		
Yes	76	31.4%
No	166	68.6%

Note. Percentages may not add up to 100% due to a small amount of missing data.

Daily Measures and Descriptive Statistics by Sample

Relationship satisfaction was assessed with one item adapted from the Perceived Relationship Quality Components (PRQC) Inventory (Fletcher et al., 2000) to ask about that day: "How satisfied were you with your relationship?" (1="not at all" to 7="extremely"; Sample 1: *M*=6.13, *SD*=1.17; Sample 2: *M*=6.04, *SD*=1.25)**.** ***Sexual satisfaction*** was assessed with five items from the Global Measure of Sexual Satisfaction (GMSEX; Lawrance & Byers, 1995) adapted to the daily context and rated on a semantic differential scale from 1 to 7 (e.g., "bad" to "good"; Sample 1: *M*=6.43, *SD*=0.90, *Rc*=.90; Sample 2: *M*=5.55, *SD*=1.68, *Rc*=.96). ***Sexual desire*** was assessed with one item: "I felt a great deal of sexual desire for my partner today" (1="strongly disagree" to 7 ="strongly agree"; Sample 1: *M*=4.38, *SD*=1.75; Sample 2: *M*=4.80, *SD*=1.82).

Each day, participants were also asked "Did you and your partner have sex today? (yes/no)." In Sample 1, participants reported engaging in sex with their partner on a total of 830 days (18%; *Range*=0 to 17 days; *M*=3.47; *SD*=3.00), and 107 couples from this sample were included in our analyses because fifteen couples did not have sex during the diary. In Sample 2, participants reported engaging in sex with their partner on a total of 1,107 days (25%; *Range*=0 to 17; *M*=4.59; *SD*=3.13), and 118 couples from this sample were included in our analyses because three couples did not have sex during the diary. Each day participants reported engaging in sex, ***amplifying sexual desire*** was assessed with an item adapted from Côté and Morgan (2002): "During sex, I tried to enhance or exaggerate my display of sexual desire" (1="strongly disagree" to 7="strongly agree"; Sample 1: *M*=2.35, *SD*=1.68; Sample 2: *M*=2.76, *SD*=1.92); ***suppressing sexual disinterest*** was assessed with an item adapted from Gross and John (2003): "When I felt disinterested during sex, I was careful not to express this" (1="strongly disagree" to

7="strongly agree"; Sample 1: *M*=2.58, *SD*=1.75; Sample 2: *M*=2.51, *SD*=2.01); and in Sample 2 only, ***sexual authenticity*** was assessed with one item adapted from Impett et al. (2012) to the sexual context: "I felt authentic (true to myself) during sex" (1="strongly disagree" to 7="strongly agree"; *M*=6.16; *SD*=1.15).

Table S3

Integrative Data Analysis Results: Main Effects of Desire Regulation During Sex on Daily Sexual Satisfaction (Random Slope Modelled for Actor Suppression)

	Sexual Satisfaction				
	b	*SE*	*t*(df)	*p*	*CI*
**Within-Person Effects**					
Actor Amplify Sexual Desire	-.05	.01	-3.31(1179.31)	.001	[-.08, -.02]
Actor Suppress Sexual Disinterest	-.03	.02	-1.63(98.82)	.11	[-.07, .01]
Partner Amplify Sexual Desire	-.02	.01	-1.13(1155.69)	.26	[-.04, .01]
Partner Suppress Sexual Disinterest	-.01	.01	-.61(1125.48)	.54	[-.04, .02]
**Sample Effects**					
Sample	.03	.06	.44(196.86)	.66	[-.09, .15]
Sample X Actor Suppress Sexual Disinterest	-.04	.02	-1.91(91.52)	.060	[-.08, .002]
Sample X Partner Suppress Sexual Disinterest	-.03	.01	-2.16(1119.53)	.031	[-.05, -.003]
**Between-Person Effects**					
Actor Amplify Sexual Desire	-.02	.04	-.61(361.40)	.55	[-.10, .05]
Actor Suppress Sexual Disinterest	-.16	.04	-4.08(351.31)	<.001	[-.24, -.08]
Partner Amplify Sexual Desire	-.07	.04	-1.72(360.56)	.086	[-.15, .01]
Partner Suppress Sexual Disinterest	-.03	.04	.44(196.86)	.66	[-.09, .15]

Note. Amplify Sexual Desire=amplify sexual desire during sex. Suppress Sexual Disinterest=suppress sexual disinterest during sex. Sample effect coded as 1=Sample 1, -1=Sample 2.

Table S4

Sample 1 Correlations Among Study Variables (n = 244)

Variable	1.	2.	3.	4.	5.	6.
1. Daily sexual desire	**.44***					
2. Daily desire amplification during sex	-.27*	**.24***				
3. Daily disinterest suppression during sex	-.21*	.56*	**.15***			
4. Daily relationship satisfaction	.53*	-.23*	-.25*	**.48***		
5. Daily sexual satisfaction	.48*	-.30*	-.30*	.49*	**.34***	
6. Gender	.23*	-.12	-.07	-.03	.08	--

Sample 2 Correlations Among Study Variables (n = 242)

Variable	1.	2.	3.	4.	5.	6.	7.
1. Daily sexual desire	**.33***						
2. Daily desire amplification during sex	.02	**.42***					
3. Daily disinterest suppression during sex	-.07	.67*	**.26***				
4. Daily relationship satisfaction	.41*	-.14*	-.19*	**.52***			
5. Daily sexual satisfaction	.46*	-.21*	-.20*	.49*	**.58***		
6. Daily sexual authenticity	.35*	-.22*	-.34*	.37*	.55*	**.33***	
7. Gender	.21*	.00	.03	-.03	-.01	-.02	--

Note. Gender coded as 0=women, 1=men. Partner correlations bolded on the diagonal. Daily variables aggregated across the course of the diary. *$p < .05$.

Sexual Authenticity: Alternative Mediation Models

Following a similar procedure employed by English and John (2013) in their study on suppression, authenticity, and social functioning, we tested two alternative mediated-moderation models for each of the original dependent variables to rule out alternative pathways.

First, we tested whether satisfaction might mediate the link between the desire regulation during sex and sexual desire interaction term and sexual authenticity (i.e., desire regulation during sex X sexual desire → satisfaction → sexual authenticity). When we tested relationship satisfaction as the mediator and sexual authenticity as the dependent variable (i.e., amplification during sex X sexual desire and suppression during sex X sexual desire → relationship satisfaction → sexual authenticity), we found that the interaction between amplification and desire predicted relationship satisfaction (b=.04, SE=.02, p=.022) such that at low levels of desire, amplification was not linked to relationship satisfaction (b=-.07, SE=.03, p=.130), but at high levels of desire, amplification was linked to marginally higher relationship satisfaction (b=.03, SE=.02, p=.080). Relationship satisfaction, in turn, was linked to higher sexual authenticity (b=.10, SE=.05, p=.037), but the indirect effect was non-significant: 95% CI of indirect effect [-.0001, .0091]. For suppression, neither the interaction between suppression and desire (b=.01, SE=.01, p=.324) nor the main effect of suppression (b=.00, SE=.02, p=.843) predicted relationship satisfaction, so we did not proceed with testing the full (moderated) mediation.

When we tested sexual satisfaction as the mediator and sexual authenticity as the dependent variable (i.e., amplification during sex X sexual desire and suppression during sex X sexual desire → sexual satisfaction → sexual authenticity), we found that the interaction between amplification and desire predicted sexual satisfaction (b=.04, SE=.02, p=.035) such that at low

levels of desire, amplification was linked to lower sexual satisfaction (*b*=-.14, *SE*=.05, *p*=.004), but at high levels of desire, this negative link was marginally weaker (*b*=-.04, *SE*=.02, *p*=.064). Sexual satisfaction, in turn, was linked to marginally higher sexual authenticity (*b*=.07, *SE*=.04, *p*=.081), but the indirect effect was non-significant: 95% CI of indirect effect [-.0004, .0070]. For suppression, neither the interaction between suppression and desire (*b*=.00, *SE*=.02, *p*=.988) nor the main effect of suppression (*b*=.02, *SE*=.02, *p*=.309) predicted sexual satisfaction, so we did not proceed with testing the full (moderated) mediation.

Next, we tested whether the interaction between desire regulation during sex and sexual desire might mediate the link between sexual authenticity and satisfaction (i.e., sexual authenticity → desire regulation during sex X sexual desire → satisfaction). When we tested sexual authenticity as the independent variable in the model predicting relationship satisfaction (i.e., sexual authenticity → amplification during sex X sexual desire and suppression during sex X sexual desire → relationship satisfaction), we found that sexual authenticity predicted lower amplification (*b*=-.15, *SE*=.05, *p*=.002). The interaction between amplification and desire, in turn, predicted relationship satisfaction (*b*=.03, *SE*=.02, *p*=.042) such that when desire was low, amplification was not linked to relationship satisfaction (*b*=-.05, *SE*=.04, *p*=.222), but when desire was high, amplification was linked to marginally higher relationship satisfaction (*b*=.03, *SE*=.02, *p*=.063). The indirect effect was significant (95% CI of the indirect effect [-.0115, -.0001]). Sexual authenticity also predicted lower suppression (*b*=-.24, *SE*=.05, *p*<.001), but neither the interaction between suppression and desire (*b*=-.01, *SE*=.01, *p*=.323) nor the main effect of suppression (*b*=.01, *SE*=.02, *p*=.644) predicted relationship satisfaction. As such, we did not proceed with testing the full (moderated) mediation.

When we tested sexual authenticity as the independent variable in the model predicting

sexual satisfaction (i.e., sexual authenticity → amplification during sex X sexual desire and suppression during sex X sexual desire → sexual satisfaction), we found that sexual authenticity predicted lower amplification (*b*=-.15, *SE*=.05, *p*=.002). The interaction between amplification and desire, in turn, marginally predicted sexual satisfaction (*b*=.03, *SE*=.02, *p*=.059) such that when desire was low, amplification was linked to lower sexual satisfaction (*b*=-.12, *SE*=.05, *p*=.009), but when desire was high, this negative link was marginally weaker (*b*=-.04, *SE*=.02, *p*=.069). The indirect effect, however, was non-significant: 95% CI of the indirect effect [-.0118, .0002]. Sexual authenticity also predicted lower suppression (*b*=-.24, *SE*=.05, *p*<.001), but neither the interaction between suppression and desire (*b*=.00, *SE*=.02, *p*=.958) nor the main effect of suppression (*b*=.03, *SE*=.02, *p*=.290) predicted sexual satisfaction. As such, we did not proceed with testing the full (moderated) mediation.

In summary, the non-significant mediated-moderation models when sexual authenticity was tested as the dependent variable provide further support for our conceptual model with satisfaction as the core outcome variable. In addition, although we did find a significant indirect effect when we tested amplification mediating the link between sexual authenticity and relationship satisfaction, we did *not* find a significant indirect effect when we tested suppression as a mediator in this model. Importantly, this contrasts with our mediation model in the manuscript, in which we found evidence for the indirect effect of both amplification *and* suppression through sexual authenticity on relationship satisfaction. We recognize that causal claims cannot be made given our cross-sectional data, and it is possible that there are bidirectional links between our core constructs—perhaps especially between amplification and sexual authenticity. Taken together, however, we do have more confidence in our proposed mediation model in the manuscript given that we generally ruled out these alternative models.

Follow-Up Analyses (Combined Samples)

Across both samples, we also examined if the chronic use of desire regulation strategies during sex over the 21-day diary period was associated with relationship satisfaction, commitment, and thoughts of breaking up three months later (as well as sexual satisfaction only in Sample 2).

Method

Person-Level Measures

Of the 243 couples across both samples, the vast majority (87%, *n*=422) completed the follow-up survey. In the baseline and follow-up surveys, participants completed measures of relationship satisfaction and commitment, and in the follow-up survey, they reported thoughts about breaking up. ***Relationship satisfaction*** was assessed with three items (e.g., “How satisfied are you with your relationship?”) from the Perceived Relationship Quality Component Inventory (PRQC; Fletcher et al., 2000) on a scale from 1=“not at all” to 7=“extremely” (Sample 1 baseline: *M*=6.20; *SD*=.93, α=.93; Sample 1 follow-up: *M*=5.95, *SD*=1.29, α=.98; Sample 2 baseline: *M*=6.14, *SD*=.92, α=.95; Sample 2 follow-up: *M*=5.97, *SD*=1.16, α=.94; Combined baseline: *M*=6.17, *SD*=.93, α=.94; Combined follow-up *M*=5.96, *SD*=1.23, α=.96). ***Commitment*** was measured with three items (e.g., “How committed are you to your relationship?”) from the PRQC (Fletcher et al., 2000) on a scale from 1=“not at all” to 7=“extremely” (Sample 1 baseline: *M*=6.65, *SD*=.79, α=.95; Sample 1 follow-up: *M*=6.46; *SD*=1.01, α=.96; Sample 2 baseline: *M*=6.69, *SD*=.59, α=.83; Sample 2 follow-up: *M*=6.58; *SD*=.86; Combined baseline: *M*=6.72; *SD*=.70; Combined follow-up: *M*=6.55; *SD*=.93)[1]. ***Thoughts about breaking up*** were assessed

[1] Although Sample 1 originally had three baseline and follow-up commitment items, Sample 2 had two baseline commitment items and one follow-up commitment item from the PRQC. To run an IDA with both samples combined, only the one-item face valid measure of commitment that was consistent across both samples was used at baseline and follow-up (i.e., “How committed are you to your relationship?”).

with four items (Booth et al., 1983; Impett et al., 2010). Three items (e.g., “Have you and your partner ever seriously suggested the idea of breaking up?”) were measured on a scale from 1 to 3 (1=“never”, 2=“within the last three months”, 3=“currently”; Sample 1 follow-up: *M*=1.20, *SD*=.37; Sample 2 follow-up: *M*=1.23, *SD*=.36; Combined follow-up: *M*=1.21, *SD*=.37) and the last item (“Have you and your partner had a separation or broken up?”) was dichotomous (1=“never”, 2=“within the last three months”; Sample 1 follow-up: *M*=1.03, *SD*=.17; Sample 2 follow-up: *M*=1.03, *SD*=.18; Combined follow-up: *M*=1.03, *SD*=.17). Items were standardized before combining them into a composite measure (Sample 1 follow-up: *M*=.00, *SD*=.78, α=.78; Sample 2 follow-up: *M*=.00, *SD*=.73, α=.71; Combined follow-up: *M*=.00, *SD*=.75, α=.75).

Data Analytic Approach

Similar to the analyses in our manuscript, we pooled data from our two samples together and conducted an integrative data analysis (IDA) following Curran and Hussong’s (2009) and Hussong and colleagues’ (2013) guidelines. We used the aggregates of participants’ reports of daily sexual desire amplification and sexual disinterest suppression during sex over the course of the diary to predict relationship satisfaction, commitment, and break-up thoughts three months later (plus sexual satisfaction only in Sample 2), controlling for the same outcome at baseline. Similar to our daily models, these analyses utilize the Actor Partner Interdependence Model (APIM; Kenny et al., 2006), with separate models for each dependent variable. We first ran indistinguishable models, then tested whether gender moderated the links between desire regulation strategy and the follow-up outcome. If an effect differed by gender, we ran distinguishable models and reported the effects separately for men and women. Truncated data and code from these follow-up analyses are available on the OSF (https://osf.io/w7gnv/?view_only=1e18eb4c94424e8da8644165a70c5de8).

Results

Correlations among variables across samples are shown in Table S5 (Table S6 and S7 depict correlations by sample). Consistent across both samples, people who chronically suppressed disinterest during sex reported lower relationship satisfaction and lower commitment three months later, controlling for initial levels of relationship satisfaction and commitment, respectively (Table S8). Gender moderated the association between desire amplification and relationship satisfaction (b=-.18, SE=.09, t(301.03)=-1.99, p=.048, 95% CI [-.37, -.002]). For women, chronically amplifying desire was not linked to their relationship satisfaction (b=.06, SE=.06, t(273.15)=.94, p=.35, 95% CI [-.06, .17]), but men who chronically amplified desire reported marginally lower relationship satisfaction (b=-.12, SE=.07, t(306.98)=-1.86, p=.063, 95% CI [-.25, .01]). Gender also marginally moderated the link between chronic suppression and thoughts of breaking up (b=.12, SE=.06, t(284.13)=1.95, p=.053, 95% CI [-.001, .24]), but given that this was a marginally significant effect, we did not break down the simple effects for men and women. Finally, in Sample 2, chronic desire regulation during sex did not predict sexual satisfaction three months later. However, gender marginally moderated the link between chronic desire amplification and a partner's sexual satisfaction (b=.30, SE=.17, t(175.85)=1.73, p=.085, 95% CI [-.04, .65]). Given that this was a marginally significant effect, we do not report the simple effects for men and women.

Table S5

Combined Samples: Correlations Among Focal Variables and Follow-Up Variables (n = 486)

Variable	1.	2.	3.	4.	5.	6.	7.	8.	9.	10.	11.
1. Daily sexual desire	**.40*** **										
2. Daily desire amplification during sex	-.09†	**.36*** **									
3. Daily disinterest suppression during sex	-.14**	.61***	**.21*** **								
4. Daily relationship satisfaction	.52***	-.26***	-.26***	**.56*** **							
5. Daily sexual satisfaction	.39***	-.27***	-.22***	.57***	**.57*** **						
6. Baseline relationship satisfaction	.36***	-.17***	-.15**	.67***	.42***	**.48*** **					
7. Baseline commitment	.15**	-.16**	-.13*	.41***	.20***	.54***	**.29*** **				
8. Follow-up relationship satisfaction	.34***	-.19***	-.22***	.58***	.41***	.48***	.40***	**.60*** **			
9. Follow-up commitment	.26***	-.19***	-.21***	.32***	.32***	.29***	.49***	.63***	**.58*** **		
10. Follow-up thoughts of breaking up	-.15**	.19***	.20***	-.28***	-.27***	-.20***	-.22***	-.66***	-.44***	**.60*** **	
11. Gender	.22***	-.05	-.01	-.04	.02	-.05	-.08^	-.01	-.04	-.03	-

Note. Gender coded as 0=women, 1=men. Partner correlations bolded on the diagonal. Daily variables aggregated across the course of the diary. ****p* < .001, ***p* < .01, **p* < .05, †*p* = .081, ^*p* =.081.

Table S6

Sample 1: Correlations among Focal Variables and Follow-Up Variables (n = 244)

Variable	1.	2.	3.	4.	5.	6.	7.	8.	9.	10.	11.
1. Daily sexual desire	**.44***										
2. Daily desire amplification during sex	-.27*	**.24***									
3. Daily disinterest suppression during sex	-.21*	.56*	**.15***								
4. Daily relationship satisfaction	.53*	-.23*	-.25*	**.48***							
5. Daily sexual satisfaction	.48*	-.30*	-.30*	.49*	**.34***						
6. Baseline relationship satisfaction	.40*	-.14*	-.07	.76*	.40*	**.48***					
7. Baseline commitment	.24*	-.12^	-.12^	.53*	.28*	.58*	**.44***				
8. Follow-up relationship satisfaction	.32*	-.18*	-.18*	.56*	.36*	.46*	.47*	**.57***			
9. Follow-up commitment	.24*	-.20*	-.22*	.40*	.34*	.33*	.65*	.71*	**.48***		
10. Follow-up thoughts of breaking up	-.14†	.19*	.18*	-.26*	-.22*	-.20*	-.26*	-.68*	-.51*	**.74***	
11. Gender	.23*	-.12	-.07	-.03	.08	-.10	-.04	-.01	-.04	-.04	-

Note. Gender coded as 0=women, 1=men. Partner correlations bolded on the diagonal. Daily variables aggregated across the course of the diary. *$p < .05$, †$p = .05$, ^$p < .09$.

Table S7

Sample 2: Correlations among Focal Variables and Follow-Up Variables (n = 242)

Variable	1.	2.	3.	4.	5.	6.	7.	8.	9.	10.	11.	12.	13.	14.
1. Daily sexual desire	**.33***													
2. Daily desire amplification during sex	.02	**.42***												
3. Daily disinterest suppression during sex	-.07	.67*	**.26***											
4. Daily relationship satisfaction	.41*	-.14*	-.19*	**.52***										
5. Daily sexual satisfaction	.46*	-.21*	-.20*	.49*	**.58***									
6. Daily sexual authenticity	.35*	-.22*	-.34*	.37*	.55*	**.33***								
7. Baseline relationship satisfaction	.34*	-.18*	-.21*	.49*	.44*	.49*	**.48***							
8. Baseline sexual satisfaction	.39*	-.20*	-.26*	.31*	.65*	.43*	.34*	**.50***						
9. Baseline commitment	.26*	-.25*	-.22*	.34*	.28*	.33*	.65*	.24*	**.43***					
10. Follow-up relationship satisfaction	.38*	-.19*	-.26*	.40*	.49*	.37*	.52*	.34*	.45*	**.64***				
11. Follow-up sexual satisfaction	.38*	-.15*	-.19*	.33*	.56*	.44*	.53*	.43*	.42*	.72*	**.39***			
12. Follow-up commitment	.35*	-.18*	-.17*	.28*	.41*	.30*	.32*	.20*	.46*	.58*	.48*	**.49***		
13. Follow-up thoughts of breaking up	-.18*	.19*	.22*	-.22*	-.34*	-.18*	-.21*	-.24*	-.21*	-.63*	-.39*	-.31*	**.50***	
14. Gender	.21*	.00	.03	-.03	-.01	-.02	.01	.04	-.01	-.01	-.00	-.02	-.01	--

Note. Gender coded as 0=women, 1=men. Partner correlations bolded on the diagonal. Daily variables aggregated across the course of the diary. *$p < .05$.

Table S8

Integrative Data Analysis Results: Desire Regulation During Sex and Follow-Up Outcomes Three Months Later Across Samples

	Relationship Satisfaction					Commitment				
	b	*SE*	*t*(df)	*p*	*CI*	*b*	*SE*	*t*(df)	*p*	*CI*
Actor Amplify Sexual Desire	-.01[g]	.04	-.28(353.44)	.78	[-.10, .07]	-.02	.03	-.72(357.06)	.48	[-.08, .04]
Partner Amplify Sexual Desire	-.06	.04	-1.37(352.22)	.17	[-.14, .03]	-.05	.03	-1.62(355.93)	.11	[-.11, .01]
Actor Suppress Sexual Disinterest	-.09	.04	-2.04(339.02)	.042	[-.17, -.003]	-.08	.03	-2.58(345.05)	.010	[-.14, -.02]
Partner Suppress Sexual Disinterest	-.03	.04	-.79(335.19)	.43	[-.12, .05]	.03	.03	.87(341.55)	.39	[-.03, .08]
Baseline DV	.51	.06	8.29(362.27)	<.001	[.39, .63]	.55	.05	10.11(350.83)	<.001	[.44, .66]
Sample	-.05	.06	-.78(184.77)	.44	[-.18, .08]	-.08	.05	-1.86(198.78)	.064	[-.17, .01]
Sample X Baseline DV						.12	.05	2.19(354.26)	.029	[.01, .22]

	Thoughts of Breaking Up					Sexual Satisfaction (Sample 2 Only)				
	b	*SE*	*t*(df)	*p*	*CI*	*b*	*SE*	*t*(df)	*p*	*CI*
Actor Amplify Sexual Desire	.04	.03	1.37(316.01)	.17	[-.02, .10]	-.07	.08	-.86(192.93)	.39	[-.23, .09]
Partner Amplify Sexual Desire	.03	.03	1.04(312.89)	.30	[-.03, .09]	.09[m]	.08	1.10(192.97)	.27	[-.07, .25]
Actor Suppress Sexual Disinterest	.05[m]	.03	1.82(298.30)	.070	[-.004, .11]	.00	.08	.03(190.70)	.97	[-.16, .16]
Partner Suppress Sexual Disinterest	-.00	.03	-.08(292.24)	.93	[-.06, .06]	-.05	.08	-.65(189.83)	.52	[-.20, .10]
Baseline DV	-.16	.05	-3.23(293.05)	.001	[-.25, -.06]	.47	.08	6.18(181.12)	<.001	[.32, .62]
Sample	.04	.05	.76(181.90)	.45	[-.06, .13]					
Sample X Baseline DV	-.12	.05	-2.41(297.86)	.017	[-.21, -.02]					

Note. DV=dependent variable. Baseline DV is commitment in the thoughts of breaking up model. [g]=effect was moderated by gender. [m]=effect was moderated by gender (marginal). Sample coded as Sample 1=1, Sample 2=-1.

Follow-Up Analyses: Summary and Discussion

Consistent across samples, we found that people who chronically suppressed disinterest during sex over the course of the diary reported lower relationship satisfaction and commitment three months later. These findings generally align with research suggesting that the habitual use of suppression is linked to higher negative emotions (Gross & John, 2003) and that deceptive affection may provide temporary fixes to relational or sexual challenges, but that lower well-being may manifest more strongly over time (e.g., Denes et al., 2017). In contrast, chronic desire amplification during sex was not associated with follow-up sexual and relational outcomes. Although desire amplification during sex was linked to lower daily sexual satisfaction (for both partners) and lower daily relationship satisfaction through feelings of sexual inauthenticity on low sexual desire days, it does not appear to have longer lasting implications for partners' relationship and sex lives.

References

Booth, A., Johnson, D., & Edwards, J. N. (1983). Measuring marital instability. *Journal of Marriage and the Family, 45*, 387-394. https://doi.org/10.2307/351516

Côté, S., & Morgan, L. M. (2002). A longitudinal analysis of the association between emotion regulation, job satisfaction, and intentions to quit. *Journal of Organizational Behavior, 23,* 947-962. https://doi.org/10.1002/job.174

Denes, A., Bennett, M., & Winkler, K. L. (2017). Exploring the benefits of affectionate communication: Implications for interpersonal acceptance-rejection theory. *Journal of Family Theory & Review, 9,* 491-506. https://doi.org/10.1111/jftr.12218

English, T., & John, O. P. (2013). Understanding the social effects of emotion regulation: The mediating role of authenticity for individual differences in suppression. *Emotion, 13,* 314-329. https://doi.org/10.1037/a0029847

Fletcher, G. J., Simpson, J. A., & Thomas, G. (2000). The measurement of perceived relationship quality components: A confirmatory factor analytic approach. *Personality and Social Psychology Bulletin, 26*, 340-354. http://dx.doi.org/10.1177/0146167200265007

Gross, J. J., & John, O. P. (2003). Individual differences in two emotion regulation processes: Implications for affect, relationships, and well-being. *Journal of Personality and Social Psychology, 85,* 348-362. https://doi.org/10.1037/0022-3514.85.2.348

Impett, E. A., Gordan, A. M., Kogan, A., Oveis, C., Gable, S. L., & Keltner, D. (2010). Moving toward more perfect unions: Daily and long-term consequences of approach and avoidance goals in romantic relationships. *Journal of Personality and Social Psychology, 99,* 948-963. https://doi.org/10.1037/a0020271

Impett, E. A., Kogan, A., English, T., John, O., Oveis, C., Gordon, A. M., & Keltner, D. (2012). Suppression sours sacrifice: Emotional and relational costs of suppressing emotions in romantic relationships. *Personality and Social Psychology Bulletin, 38,* 707-720. https://doi.org/10.1177/0146167212437249

Kenny, D. A., Kashy, D. A., & Cook, W. L. (2006). *The analysis of dyadic data*. Guilford Press.

Lawrence, K., & Byers, E. S. (1995). Sexual satisfaction in long-term heterosexual relationships: The Interpersonal Exchange Model of Sexual Satisfaction. *Personal Relationships, 2,* 267-285. https://doi.org/10.1111/j.1475-6811.1995.tb00092.x
